# A Qualitative Exploration of Stakeholders’ Preferences for Early-Stage Rectal Cancer Treatment

**DOI:** 10.1097/AS9.0000000000000364

**Published:** 2023-12-14

**Authors:** Merrill E. Rubens, Timothy P. Mayo, Radhika K. Smith, Sean C. Glasgow, Mary C. Politi

**Affiliations:** From the * Department of Surgery; † Section of Colon and Rectal Surgery, Department of Surgery; ‡ Division of Public Health Sciences, Department of Surgery, Washington University School of Medicine in St. Louis, MO.

**Keywords:** active surveillance, decision aid, rectal cancer, shared decision-making

## Abstract

As treatment options for patients with rectal cancer evolve, patients with early-stage rectal cancer may have a treatment choice between surgery and a trial of nonoperative management. Patients must consider the treatments’ clinical tradeoffs alongside their personal goals and preferences. Shared decision-making (SDM) between patients and clinicians can improve decision quality when patients are faced with preference-sensitive care options. We interviewed 28 stakeholders (13 clinicians and 15 patients) to understand their perspectives on early-stage rectal cancer treatment decision-making. Clinicians included surgeons, medical oncologists, and radiation oncologists who treat rectal cancer. Adult patients included those diagnosed with early-stage rectal cancer in the past 5 years, recruited from an institutional database. A semi-structured interview guide was developed based on a well-established decision support framework and reviewed by the research team and stakeholders. Interviews were conducted between January 2022 and January 2023. Transcripts were coded by 2 raters and analyzed using thematic analysis. Both clinicians and patients recognized the importance of SDM to support high-quality decisions about the treatment of early-stage rectal cancer. Barriers to SDM included variable clinician motivation due to lack of training or perception of patients’ desires or abilities to engage, as well as time-constrained encounters. A decision aid could help facilitate SDM for early-stage rectal cancer by providing standardized, evidence-based information about treatment options that align with clinicians’ and patients’ decision needs.

## Abstract 2

Mini abstract

As treatment options for patients with rectal cancer evolve, patients with early-stage rectal cancer may have a treatment choice between surgery and a trial of nonoperative management. Shared decision-making (SDM) between patients and clinicians can improve decision quality when patients are faced with preference-sensitive care options. We performed a qualitative study utilizing one-on-one interviews with 28 stakeholders (13 clinicians and 15 patients) to understand their perspectives on early-stage rectal cancer treatment decision-making. Both clinicians and patients recognized the importance of SDM to support high-quality decisions about the treatment of early-stage rectal cancer. Barriers to SDM included variable clinician motivation due to lack of training or perception of patients’ desires or abilities to engage, as well as time-constrained encounters. A decision aid could help facilitate SDM for early-stage rectal cancer by providing standardized, evidence-based information about treatment options that align with clinicians’ and patients’ decision needs.

## INTRODUCTION

As treatment options for rectal cancer continue to evolve, select patients with early-stage rectal cancer may be offered the choice between 2 medically appropriate treatment options: operative (surgery) or a trial of nonoperative (eg, combination of radiation therapy and chemotherapy) management. For patients with early-stage or clinical stage 1 (eg. T1-2, N0, or M0) rectal cancer not amenable to local resection, the standard of care historically has been transabdominal resection alone, which often involves a permanent or temporary ostomy. An alternative, although still experimental, treatment paradigm has recently emerged in which patients start with total neoadjuvant chemotherapy and radiation. If patients have a complete clinical response (cCR) with no evidence of tumor on repeat staging after neoadjuvant therapy, they might be spared the morbidity of surgery and instead follow a surveillance protocol to monitor for signs of cancer regrowth.^1^ This treatment paradigm gives patients better functional outcomes with similar overall survival.^2–5^ Although there is limited data on cCR following neoadjuvant chemotherapy and radiation for early-stage rectal cancer patients, studies show it is possible in about 50% of patients.^6^ Yet, there is a 20% to 25% risk of local recurrence within the first 2 to 3 years after cCR that would then require surgery.^2,4,7^ Patients and clinicians must weigh the morbidities of surgery against those of neoadjuvant chemotherapy and radiation, as well as the fact that neoadjuvant chemotherapy and radiation are not currently standard care. They must also consider the chance that the patient could still need surgery or have an increased chance of recurrence after neoadjuvant chemotherapy and radiation.

Shared decision-making (SDM) is a way for patients and clinicians to work together to reach an agreement about a health decision involving multiple medically appropriate treatment options, weighing evidence alongside patients’ preferences for possible outcomes.^8^ Most patients desire an active role in health decisions, and clinicians seek patient-centered ways to communicate the complexities of multiple reasonable treatment options.^9–11^ SDM can be enhanced through the use of high-quality decision aids. Decision aids can increase patient knowledge and understanding of risks while improving the match between personal preferences and choices.^12^ The process of SDM, often supported by decision aids, has been successfully applied to many cancer decisions with numerous decision aids available.^13–16^

We aimed to explore patients’ and clinicians’ perspectives on decision-making needs and preferences when considering treatment options for early-stage rectal cancer, including whether a decision aid would be helpful.

## METHODS

The Washington University in St Louis Human Research Protection Office approved this project as an exempt study (HRPO# 202108159). The design, conduct, and reporting of this study followed the consolidated criteria for Reporting Qualitative research guidelines.^17^ Patients were identified from an institutional, prospectively-maintained rectal cancer registry. Eligibility criteria included adult patients (over age 18) who were treated for early-stage rectal cancer (eg. T1-2, N0, or M0) within the past 5 years. All eligible patients underwent surgery alone or neoadjuvant chemotherapy and radiation followed by either surgery or active surveillance, per institutional protocol. Potential participants were contacted via telephone to confirm eligibility and complete consent before interviews. Purposive sampling^18^ was used to obtain a range of patients with different treatment histories, gender, and age.

Clinician recruitment began by contacting rectal cancer clinicians at one institution. Recruitment continued through snowball sampling^19^ where we asked each participating clinician to identify other eligible clinicians nationwide. Clinician inclusion criteria were: colorectal surgeons, medical oncologists, and radiation oncologists who treat rectal cancer patients where nonoperative management might be pursued for early-stage rectal cancer. Treating early-stage rectal cancer with neoadjuvant chemotherapy and radiation rather than surgery is not yet recommended in all medical centers. We aimed to enroll clinicians from different institutions to account for regional or institutional differences in practice.

Participants were enrolled until thematic saturation was reached.^20^ Interviews took place via telephone. No repeat interviews were conducted. All interviews were conducted by a research fellow trained in qualitative research methods. The interviewer was not involved in the treatment of participants but understood the disease and decision complexity. Participants were told that study findings would be used to develop a decision aid to assist future patients. Interviews were audio-recorded with participants’ consent.

The interview guide (Appendix A, http://links.lww.com/AOSO/A283) was constructed based on the Ottawa Decision Support Framework (ODSF).^21^ Patient interviews explored issues that were important during the decision-making process, who was involved in the process and the challenges they faced. Participants were also asked if they thought a patient decision aid would have helped in the decision process and, if so, what format would have been most helpful (paper-based, electronic, or video-based). Clinician interviews addressed the same broad topics but focused on how clinicians counsel patients through such decisions, whether treatment conversations differ based on clinical or patient characteristics and the best time to use a decision aid. Interviews lasted approximately 20 to 30 minutes (range 8–31 minutes). Patient participants received a $10 gift card. Clinician participants were not compensated monetarily.

Interviews were professionally transcribed. A preliminary codebook was developed and updated after coding 1–3 interviews. Transcripts were coded by 2 coders trained in qualitative interviews. Coders read all transcripts to gain an understanding of the data and identify major themes. Using the constant comparative method, each investigator separately coded the first 3 transcripts and compared the agreement. Discrepancies were discussed to reach a consensus. If consensus could not be reached, they consulted a third research team member. This process continued until inter-rater reliability was achieved (kappa ≥0.75 and ≥95% agreement on each code and overall). Inter-rater reliability was reached after 7 transcripts; investigators then separately coded the remaining 21 transcripts, meeting regularly to discuss them. Coded data was grouped into categories, and themes were identified for further analysis using the ODSF.

## RESULTS

Twenty-five patients and 16 clinicians were approached. Four patients declined, 5 could not be reached, and 1 patient and 3 clinicians agreed but later could not be reached. Twenty-eight interviews were conducted (15 patients and 13 clinicians) from January 2022 to January 2023. Table 1 describes the sample characteristics. Of note, the database overall is composed of 63% male, 91% white, and 8% black patients, but only a small percentage of patients had early-stage rectal cancer and were eligible for our study. The main themes that appeared in the data are described below along with illustrative quotes (P = patient, C = clinician).

**TABLE 1.** Participant Characteristics

Characteristics	Number (%)
Patient age at diagnosis	
30–50	4 (27%)
51–70	8 (53%)
71–90	3 (20%)
Patient self-reported dender	
Female	7 (47%)
Male	8 (53%)
Patient race*	
White	15 (100%)
Patient treatment received	
Surgery only	5 (33%)
Neoadjuvant chemotherapy and radiation followed by active surveillance	6 (40%)
Neoadjuvant chemotherapy and radiation followed by surgery	4 (27%)
Highest level of schooling	
High school diploma, GED or less	2 (13%)
Some college or technical training	3 (20%)
College degree or higher	8 (53%)
Missing	2 (13%)
Self-reported physical health status	
Poor	0 (0%)
Fair	0 (0%)
Good	4 (27%)
Very good	6 (40%)
Excellent	2 (13%)
Missing	3 (20%)
Clinician self-reported gender	
Female	6 (46%)
Male	7 (54%)
Clinician’s United States region of practice	
Northeast	1 (7.7%)
Midwest	11 (84.6%)
South	1 (7.7%)
Clinician’s practice type	
Academic	13 (100%)

* Lack of nonwhite patients available in the database who met inclusion criteria.

### Theme 1: Most Clinicians and Patients Recognized the Importance of Shared Decision-Making to Support High-Quality Decisions in this Clinical Context

Several clinicians demonstrated a thorough understanding of the components of SDM and emphasized the importance of using SDM to discuss treatment options for early-stage rectal cancer. Patients appreciated when their clinicians presented options and framed the decision in terms of patients’ values and goals of care.

*“I try to [use SDM] as much as possible…[I] try to get a better appreciation for where they [patients] are in terms of the medical knowledge, in terms of their appreciation of their circumstances, in terms of their goals.” [C22, surgeon]*.

*“I always try to walk through what matters for that individual patient and what long-term quality-of-life factors would be tolerable or intolerable to them…You need to figure out what their values are, and then you need to help them draw out how each of the choices might impact those things that they value so that then they can pick what actually is best in keeping with their values.” [C20, medical or radiation oncologist]*.

*“Dr. [Surgeon]…introduced himself as my quarterback, and I really appreciated that. He presented all those options…It was all…what was best for me…it put me in a position where I was confident in whatever I chose was going to be an okay decision for me.” [P2, chose surgery]*.

Patients agreed that more information about each treatment option could help them clarify their preferences.

*“…having more facts…having more information [helps]… it’s just hard to know what to do… I think what probably turned me around to go this route [non-operative management]…was just finally getting some information.” [P11, chose neoadjuvant chemotherapy + radiation followed by active surveillance]*.

Clinicians acknowledge that patients’ experiences and baseline knowledge can influence their choices. SDM can give clinicians better insight into their patients’ decision-making process.

*“I would also generally take the patient’s temperature on their experience, either personally or family…if they had a family member that had a lot of struggles with undergoing chemotherapy or radiation…[or] if they had…a family member who had an ostomy for an unrelated reason, and really struggled with that…you sort of meet them where they are.” [C5, surgeon]*.

*“Patients…may have had a family member who didn’t do well with chemotherapy, so they are a little hesitant to pursue it. And they want to know why they can’t just go directly to surgery…And then there are some who prefer not to have surgery altogether.” [C13, medical or radiation oncologist]*.

### Theme 2: Despite Its Importance, There are Several Challenges to Engaging in SDM for Early-Stage Rectal Cancer

Some clinicians misunderstood SDM and perceived it to be more like patient-centered communication with a recommendation, rather than bidirectional deliberation with the patient about their preferences.

*“I would say I almost always engage in shared decision making with these patients. Explaining all the potential options that we have. Why we would do certain things and understanding the rationale behind them. And then offering both my professional opinion as well as how things really fit in with [Institutional] guidelines and national guidelines.” [C24, surgeon]*.

The time-pressured nature of clinical encounters was discussed frequently as a barrier to SDM by both patients and clinicians.

*“You try to learn as quickly about them as you can. You have your consult time slots and not loads of time to figure out: What is it that they do? What is it that they value in their life?” [C20, medical or radiation oncologist]*.

*“I think these are some of the most complicated conversations that we have with patients. And obviously, we’ve got a limited amount of time to do it.” [C22, surgeon]*.

*“It happened so fast, it was a lot to process…I just went to the appointments…and I didn’t really think a whole lot…But of course when you’re in that situation…just hearing that word ‘cancer’ is the scariest word I think you can hear…and so you’re kind of numb, and there’s so much information that…sometimes you don’t process it all” [P6, chose neoadjuvant chemotherapy + radiation followed by active surveillance]*.

Some clinicians expressed concern that patients’ health literacy might impact their ability to engage in the decision-making process.

*“You’re explaining a lot of very complex things. And there’re some patients who are just like, ‘Man, Doctor, I don’t want to hear all that stuff. Just tell me what I should do’… health literacy I think is just one of the biggest barriers… this stuff is nuanced. And it is really difficult to get across…exactly all the different options and all the different repercussions of those options.” [C24, surgeon]*.

Other clinicians expressed fear that SDM might not be practiced by all members of the treatment team, leading to confusion.

*“One of the hardest things in this is that medical oncology talks to them, radiation oncology talks to them, and surgery talks to them. And they [can] get different flavors from each one of those disciplines… Those are all unique perspectives that everybody else brings to it that often times, confuse the patient….” [C3, surgeon]*.

### Theme 3: Decision Aids Might Help Patients and Clinicians Supplement Clinical Discussions and Support High-Quality Early-Stage Rectal Cancer Treatment Decisions

Most patients and clinicians interviewed agreed that a decision aid would be helpful in this context. Decision aids provide standard, accessible information and can help patients process information and think through their options.

*“I think that [a decision aid] would’ve been helpful…I probably still would’ve overthought it. But…having those really…common things that people think about in one place would be good” [P2, chose surgery]*.

*“Having more information readily available for patients…would be very helpful. I know oftentimes patients will express…that they’re overwhelmed and don’t know what questions to ask.…being able to have a decision aid and something you can potentially go through with them, something that they can read that serves as a resource…I think would be incredibly valuable.” [C18, surgeon]*.

*“They can have it written out, go home, think about it, discuss it with their family, and be able to look at something very clear and concise.” [C23, surgeon]*.

Decision aids can amplify evidence-based information and minimize misinformation or lower-quality information that may be readily available to patients.

*“People look things up, and I’m not always able to…make sure they use good resources or verified resources.” [C25, surgeon]*.

*“I had joined a [social media] group and I actually had a woman reach out to me…And she was like, ‘Hey, I’ve been…in this group for a long time. And I’m going to tell you now to search for things specifically that you’re looking for, but don’t ask for advice because everybody in here has horror stories. And it’s going to scare the shit out of you.’ …And I…appreciated that so much.” [P2, chose surgery]*.

There was no universally agreed upon optimal format for the decision aid(s), but several suggested a combination of paper and electronic versions.

*“You really do have to meet everyone where they are and have access to both options [paper and electronic]. But I do think having a paper copy is important…anything you have in your hand, you can make digital.” [C5, surgeon]*.

*“It has to accommodate everybody. And so some patients are just pencil and paper type of people…and…having a sheet of paper that you can go through with them right then and there or hand them and then draw on it yourself to augment a couple points I think is very valuable. [But] we’re seeing more and more young patients who are more technologically savvy. And so having it in electronic form they can read before the appointment and after the appointment, you know, carry with them, pull up on their phone I think could also be very valuable.” [C18, surgeon]*.

Participants suggested that the content of decision aids and conversations about surgery for early-stage rectal cancer should include the key points found in Supplemental Table 1, http://links.lww.com/AOSO/A278. Many patients shared concern over the need for a long-term or short-term ostomy. This was the most commonly noted deterrent to choosing surgery, along with general concerns about undergoing a surgical procedure. Other less commonly noted concerns were changes to sexual and urinary function, bleeding, infection, hernia, and ileus.

Considerations related to undergoing a trial of nonoperative management with chemotherapy and radiation can be found in Supplemental Table 2, http://links.lww.com/AOSO/A279. Many patients who had undergone chemotherapy and radiation talked about their experiences with neuropathy, fatigue, and short-term radiation proctitis. Some mentioned long-term changes to their bowel function after radiation therapy. Less commonly mentioned concerns included cold sensitivity, hand-and-foot syndrome, cytopenia, and vaginal stenosis.

Considerations that were pertinent to those who chose chemotherapy and radiation and were subsequently found to be candidates for active surveillance can be found in Supplemental Table 3, http://links.lww.com/AOSO/A280. Several clinicians noted that they consider patients’ ability to implement a strict follow-up regimen before offering nonoperative management due to the high frequency of examinations and the fear that a cancer regrowth could go untreated if not closely monitored. Patients expressed the emotional burden of undergoing frequent testing with a fear of recurrence.

**FIGURE 1.**
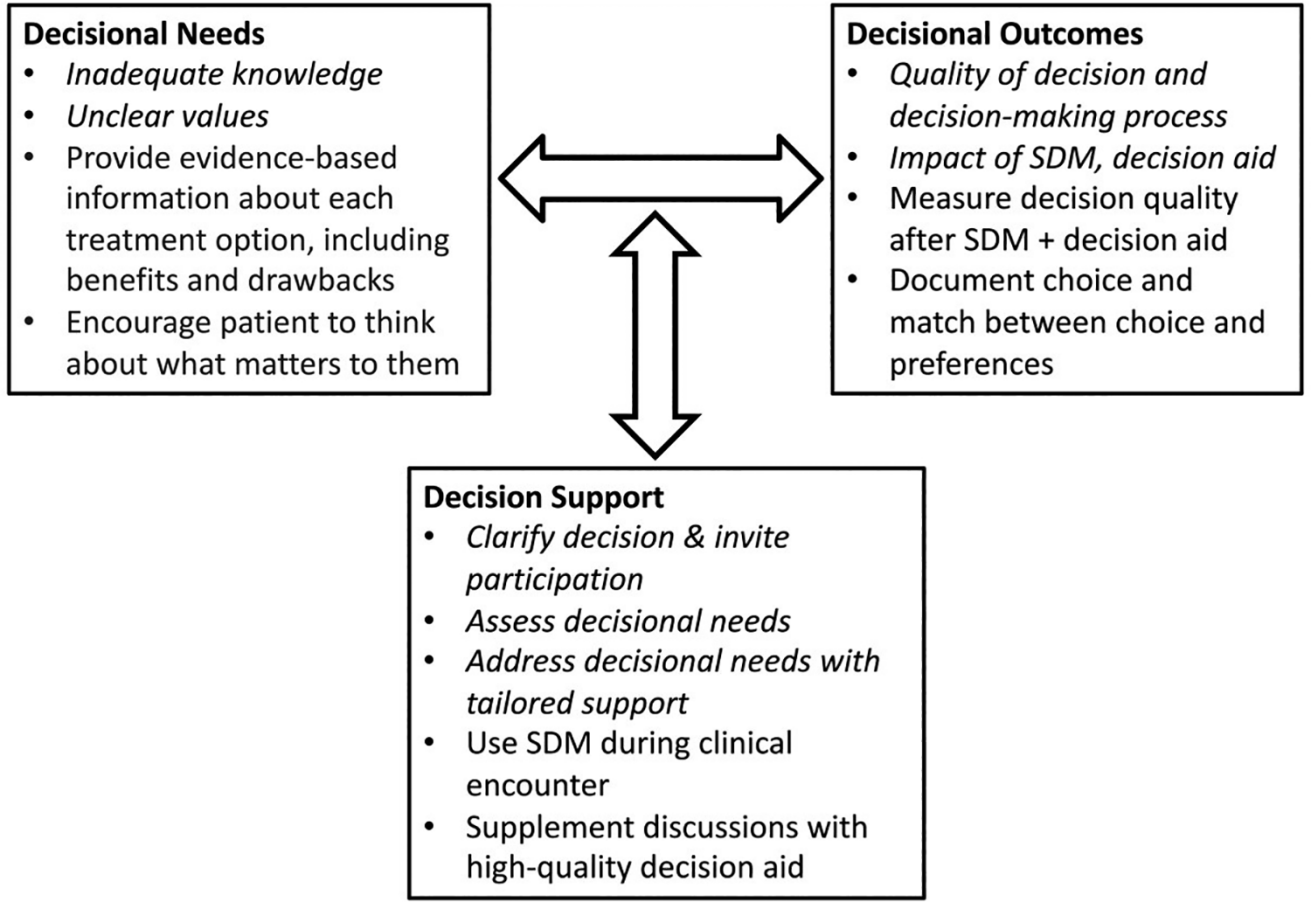
Ottawa Decision Support Framework (italicized) with study findings categorized within the framework.

There were also concerns related to both treatment options, which can be found in Supplemental Table 4, http://links.lww.com/AOSO/A281. The chance of survival was mentioned by some patients. A minority of patients were concerned about the direct treatment costs and indirect costs of unpaid leave from work. The lack of transparency in medical costs was also noted. Figure 1 displays important themes uncovered by participants, organized within the ODSF framework.

## DISCUSSION

Overall, clinicians and patients highlighted the importance of SDM about treatment options for early-stage rectal cancer. Most clinicians stated that they use SDM in this context often, recognizing that patient preferences should guide treatment decisions. Other modes of communication included patient-centered care conversations where providers explained the treatment options and led the decision but did not elicit patients’ goals or preferences or engage them in collaborative decision-making. Patients noted that more information and discussion about each treatment option would be helpful in clarifying their preferences, even if presenting the information in a single clinic visit could be overwhelming.

Despite its importance, multiple barriers exist to practicing high-quality SDM in clinical practice. A few clinicians misunderstood SDM and perceived it to be more like patient-centered communication with a recommendation rather than purposeful bidirectional deliberation with the patient about how treatment options align with their preferences. Other clinicians worried about the inconsistent use of SDM by other members of the treatment team. Both of these concerns could be addressed by providing clinicians with more education and training in SDM techniques^22^ or high-quality conversation-based decision aids to standardize communication about options.^23^ Another common concern was the time requirement of practicing SDM in an already time-pressured setting, a concern that has been reported in other studies.^24,25^ A minority of clinicians felt that patients with limited education or health literacy are not able or do not desire to participate in SDM. However, past research shows that these factors are not clear predictors of patients’ desire to engage in SDM,^26,27^ and patients across literacy levels can use effective and accessible decision tools.^28^

A decision aid for early-stage rectal cancer may address many of these barriers. Decision aids can increase patient knowledge as well as the match between values and treatment choices.^12^ They can provide standardized information to patients about their cancer care, compensating for differences in information provided between clinicians or even between visits with the same clinician. They can be purposively designed to meet the needs of people across literacy levels^29^ and reviewed by community and clinician stakeholders for clarity and accuracy. Decision aids can add a small amount of time (~2–3 minutes) when used during consultations but often no added time when the decision aid is distributed before consultations.^12^ It is possible that a decision aid for early-stage rectal cancer could reduce consultation length (considering the combined consultation time of surgery, medical oncology, and, radiation oncology) by allowing for patient education and deliberation outside of the clinic. Furthermore, as noted by clinicians and patient participants, the considerations of treatment options for early-stage rectal cancer are not only numerous but complex. Only a minority of patients reported asking about concerns such as differences in survival rate or cost between treatment options. This may be because patients feel uncomfortable talking about these topics with their treatment team or because they do not think to ask. A decision aid may more explicitly communicate some of the most complex considerations, such as the potential need for surgery after neoadjuvant chemotherapy and radiation, the reasoning behind strict surveillance compliance after a cCR, a detailed surveillance schedule, the increased risk of recurrence (and associated emotional burden) among patients undergoing a trial of nonoperative management, and the limitations of the current data that exists for nonoperative management.

This study should be interpreted within the context of some key limitations. Qualitative results do not necessarily reflect the thoughts and experiences of all early-stage rectal cancer patients and clinicians because the goal of qualitative research is to explore in-depth perspectives about a topic rather than obtain generalizable information. Patients were recruited from a single, academic institutional database that might not represent the experiences of patients at nonacademic institutions or institutions in other regions. The racial diversity of patients was limited by racial diversity in the database as a whole, as well as those presenting with early-stage rectal cancer. Of note, this does not reflect presentation with early-stage colorectal cancer on a population level (37% non-Hispanic white *vs* 35% non-Hispanic black),^30^ but instead likely reflects those seeking care at a large academic institution. Clinician selection was limited in that there are few centers in the United States currently offering the option for neoadjuvant chemotherapy and radiation and potential nonoperative management for early-stage rectal cancer patients.

Our results suggest that a decision aid to support SDM may enhance decision quality among those making a decision about early-stage rectal cancer treatment. To our knowledge, no other tools or resources exist for this particular decision scenario, since this treatment paradigm for early-stage rectal cancers is not yet widely practiced. It could lend structure to patient-clinician discussions and summarize complex information so that patients with early-stage rectal cancer can make a high-quality, preference-consistent decision about treatment.

## ACKNOWLEDGMENTS

The authors wish to thank all the individuals who gave their time and thoughtfulness toward our interviews. This publication was supported by the Washington University School of Medicine Surgical Oncology Basic Science and Translational Research Training Program grant T32 CA009621 from the National Cancer Institute (NCI) and by the Center for Collaborative Care Decisions, funded by the Foundation for Barnes Jewish Hospital (award #5799). The content is solely the responsibility of the authors and does not necessarily represent the official views of the NIH.

## Supplementary Material


