# Supplementary material for: A Qualitative Exploration of Stakeholders’ Preferences for Early-Stage Rectal Cancer Treatment
Source: Ann Surg Open. 2023 Dec 14;4(4):e364. doi: 10.1097/AS9.0000000000000364 (PMC10735060; doi:10.1097/AS9.0000000000000364)
Supplement: Supplementary file 1 [file as9-4-e364-s001.pdf]

Supplemental Table 1. Considerations related to surgical management

Potential need for ostomy, long-term or temporary	"They're…wearing this colostomy bag for the rest of their lives. Which, you know, is a huge emotional [and] physical challenge for these patients" [C7, medical or radiation oncology]<br>"I did not want a colostomy bag… My grandfather had colon cancer... And I remember the bag…so that was probably the biggest fear." [P2, chose surgery]
Risk of undergoing surgical procedure	"That was mostly pretty obvious to at least not go with surgery if it was avoidable… Surgery itself I think is always a risk of course." [P1, chose neoadjuvant chemotherapy + radiation followed by active surveillance]<br>"Well, I think any rational person would try to avoid surgery if they possibly could." [P26, neoadjuvant chemotherapy and radiation followed by surgery]<br>"What I've found is that the vast majority of patients are more pleased with the option of no surgery, rather than the possible risk that the cancer could be persistent and/ or recur. This seems like by far and away their biggest concern is, 'Do I have to have surgery?'" [C19, medical or radiation oncology]
Other	"[I] certainly talk to them about…sexual and urinary function. Certainly with anastomoses…risk of leak being probably the most important one that I'll spend the most time on." [C18, surgeon]<br>"Bleeding, infection, anastomotic complications, bowel [and] bladder dysfunction, hernias…what to expect from your change in bowel habits." [C3, surgeon]<br>"Nobody told me there was gonna be blood. I passed a lot of blood. And then I developed what's called an ileus, where my colon just…shut down, like it quit working…I started vomiting." [P27, chose surgery]
