# Supplementary material for: A Qualitative Exploration of Stakeholders’ Preferences for Early-Stage Rectal Cancer Treatment
Source: Ann Surg Open. 2023 Dec 14;4(4):e364. doi: 10.1097/AS9.0000000000000364 (PMC10735060; doi:10.1097/AS9.0000000000000364)
Supplement: Supplementary file 2 [file as9-4-e364-s002.pdf]

Supplemental Table 2. Considerations related to trial of non-operative management with chemotherapy and radiation followed by active surveillance

**Chemotherapy**	
Neuropathy	“I still have problems with my feet…It's manageable I guess would be the way to put it. I've learned to live with it. But I still have it. And I doubt if that's probably gonna go away at this point.” [P11, chose neoadjuvant chemotherapy + radiation followed by active surveillance]<br>“I would have like neuropathy type stuff, like in my hands and my feet. My hands have pretty much come out of it, but my feet are still in it… They still feel like I’m walking on marbles, but I don’t know if that’ll ever go away. He said in some people it goes away, and some people it doesn’t.” [P14, chose neoadjuvant chemotherapy + radiation followed by active surveillance]
Fatigue/Brain Fog	“It just controlled our lives…We couldn’t go anywhere or do anything. I just was not up to it.” [P6, chose neoadjuvant chemotherapy + radiation followed by active surveillance]<br>“That chemo’s hard. It’s hard on your body. It’s hard on your mind. It made me forget things.” [P16, chose neoadjuvant chemotherapy + radiation; then needed surgery]
Other	“The whole aversion to cold was interesting” [P12, chose neoadjuvant chemotherapy + radiation followed by active surveillance]<br>“I think [all patients] need to know about…the cell count production, so cytopenias” [C13, medical or radiation oncology]<br>“I couldn’t do any food prep because…the hand-foot syndrome where my hands got red and swollen, and then they peeled like they were sunburned” [P6, chose neoadjuvant chemotherapy + radiation followed by active surveillance]

**Radiation therapy**	
Proctitis/Diarrhea	"I'll tell you what, the pain that I went through with the radiation was horrid. It was terrible. It really was, it was bad. But I'll tell you what, to save your life, and if it works, it's worth going through that. But it was pretty bad… It just felt like you was going to the bathroom and it was just like lava, hot lava coming out, felt like it was just burning you…That's what it felt like. And it was just terrible. It was like that for gee whiz, I don't even know how long it was. I think it was probably almost a month." [P14, chose neoadjuvant chemotherapy + radiation followed by active surveillance]<br>"They warned me that…probably within a week of treatment I would get, you know—be very sick as far as having problems…with my intestines and bowels...like going to the bathroom frequently. But to be honest, it was a lot more than what I anticipated." [P11, chose neoadjuvant chemotherapy + radiation followed by active surveillance]
Fecal Incontinence	"If I have to use the restroom, I need to go right then. You know, like, [my work space] is right across from the restroom, just because you just—it just is. The thing that helped—that cures you also does a number on the body, too." [P8, chose neoadjuvant chemotherapy + radiation followed by active surveillance]<br>"My rectal muscles are weak. And so, it has improved, but I do have problems with leakage… I can't hold, you know, a bowel movement. So like, when I have the feeling where I need to go to the bathroom, I do have to get a bathroom [laugh]." [P11, chose neoadjuvant chemotherapy + radiation followed by active surveillance]
Other	"There's less common things…like insufficiency fractures in the pelvis or sacrum, although those are pretty rare… And then if they're…really young, you have to start thinking about things like risk of secondary cancers." [C20, medical or radiation oncology]

	"I understand that vaginal stenosis, the drying…painful intercourse, all that, is a long-term side effect" [P6, chose neoadjuvant chemotherapy + radiation followed by active surveillance]
