# Supplementary material for: A Qualitative Exploration of Stakeholders’ Preferences for Early-Stage Rectal Cancer Treatment
Source: Ann Surg Open. 2023 Dec 14;4(4):e364. doi: 10.1097/AS9.0000000000000364 (PMC10735060; doi:10.1097/AS9.0000000000000364)
Supplement: Supplementary file 3 [file as9-4-e364-s003.pdf]

Supplemental Table 3. Considerations related to active surveillance regimen

Compliance with surveillance regimen due to frequency of required tests and exams	"They need…a…clear understanding of what it is that they're getting into… because, if they choose non-operative management, the surveillance is, often times, as hard as recovering from the surgery" [C3, surgeon]<br>"To be a good candidate for total neoadjuvant therapy then surveillance after a complete response, they would have to be reliable for a follow-up… for surveillance MRIs and endoscopies, and then…how far away they live from facilities that offer the surveillance…they require" [C25, surgeon]<br>"Well, I don't like [the follow-up tests] of course. I think they're too often. But the doctors obviously don't care what I think…Every six months I have a MRI, a PET scan, sometimes a CT. And then every year they want me to have a colonoscopy, which I think's too frequent. But…when I looked it up, it does say that's the gold standard to really make sure nothing's there." [P1, chose neoadjuvant chemotherapy + radiation followed by active surveillance]
Anxiety about cancer coming back	"I don't like the anxiety of it all…the anxiety of thinking, 'Well, maybe…they could find something. If we weren't looking we wouldn't find anything.' [Laughing]" [P1, chose neoadjuvant chemotherapy + radiation followed by active surveillance]<br>"The last two snowstorms, I was supposed to go for my sigmoidoscopy. Well, they both got postponed, and that's kind of given me a lot of anxiety… thinking, 'Oh, no, what if something and, you know, is coming back?' and they don't catch it in time. And now I've, you know, wasted another month or whatever." [P8, chose neoadjuvant chemotherapy + radiation followed by active surveillance]<br>"Then I thought, 'What if I go through all that and then I have to worry with having to check every, I don't know, three months, six months, and having that in

	the back of my mind, going, oh my gosh, did it come back? Did it come back? Did they get a—you know, did the medicine shrink it?'…That would've affected more my quality of life because I would've constantly been going, 'Ooh, I hope it's okay. I hope it's gone.' Whereas now I know it's gone." [P27, chose surgery]<br>"And so [patients] have to think about whether or not can I live with this, you know, cancer in me still or potential cancer, and am I willing to come back very frequently for imaging and flex sigs." [C23, surgeon]
