# Supplementary material for: A Qualitative Exploration of Stakeholders’ Preferences for Early-Stage Rectal Cancer Treatment
Source: Ann Surg Open. 2023 Dec 14;4(4):e364. doi: 10.1097/AS9.0000000000000364 (PMC10735060; doi:10.1097/AS9.0000000000000364)
Supplement: Supplementary file 4 [file as9-4-e364-s004.pdf]

Supplemental Table 4. Universal treatment considerations

Survival rate	“The first thing I asked was ‘What’s my survival rate?’ You know, how many people my age have you treated with…a tumor like this, and how many survived? That was my very first question.” [P8, chose neoadjuvant chemotherapy + radiation followed by active surveillance]
Cost	“How do you contact your insurance company and find out what they’re gonna cover, an estimate of what the costs are? 'Cause I was absolutely stunned when I saw the costs coming through… What the hospital was charging and what the insurance company was paying…the amounts are staggering.” [P6, chose neoadjuvant chemotherapy + radiation followed by active surveillance]<br>“Just thinking of also the financial aspects of it as far as, you know, how are we going to continue if I wasn't…able to work for a while?… I did have health insurance. It wasn't like there wasn't insurance to help pay for the treatment and that. But, just the fact of it—your health insurance doesn't cover everything in full either, so you know that you're still gonna be responsible for a portion. And then…if you're not able to work…just relying on, you know, one income.” [P11, chose neoadjuvant chemotherapy + radiation followed by active surveillance]<br>“I didn’t care about the cost, as long as everything was okay…Unlike most people, I had good insurance.” [P15, chose surgery]
