# Supplementary material for: A Qualitative Exploration of Stakeholders’ Preferences for Early-Stage Rectal Cancer Treatment
Source: Ann Surg Open. 2023 Dec 14;4(4):e364. doi: 10.1097/AS9.0000000000000364 (PMC10735060; doi:10.1097/AS9.0000000000000364)
Supplement: Supplementary file 5 [file as9-4-e364-s005.pdf]

Appendix A: Interview Guide

Patient Interview Guide:

1. Tell me about how you came to be diagnosed with rectal cancer.

2. What treatment options were presented to you?
   a. Probes: surgery, chemotherapy with IV or oral medications, radiation therapy, active surveillance/monitoring carefully with frequent check-ups or scans?

3. Who was involved in the conversation about your treatment options?
   a. Probes: surgeon, nurse, family members, patient care coordinator

4. When discussing your treatment options with your care team, what did you find helpful?

5. What issues were important to you as you made your decision about treatment? Which of these issues were the most important?
   a. Probes: recovery time, cost, cancer coming back, frequent appointments, potential of permanent colostomy, sexual dysfunction, incontinence

6. What were your biggest fears or concerns about your decision?
   a. Did you discuss your concerns with your doctor?
   b. What was the hardest part about deciding on a treatment?

7. Where did you look for information when making your decision? Was there a resource that was helpful to you as you made your decision?

8. Is there anything you know now that you wish you had known when making your decision?

9. Have you heard of the term "patient decision aid"? What have you heard about them? *[Read after participant has answered]* To make sure we are on the same page, a decision aid is a tool that is designed to help people think about their health choices. It provides information about options, helps people think about what matters to them, and then helps them make a step by step decision. Decision aids come in different formats such as paper, online, and video.
   a. Do you think using a decision aid would have helped you in making your decision? In what way?
   b. How might it hinder the decision?
   c. What do you think would be the best format for this tool? (eg paper, online, video)

10. Is there anything else you would like to add or anything you would want future patients in a similar situation to know?

Thank you so much for your time. We really appreciate this perspective, and we will use what you and others tell us to help improve the way we talk to patients about this decision.

Clinician Interview Guide:
As we talk today, I'd like you to think about your patients with early (eg. cT2, node-negative) rectal cancer.

1. How do you decide on a treatment plan for this patient, specifically total mesorectal excision vs neoadjuvant chemoradiation followed by TME or non-operative management with frequent follow-up exams?
    a. What has worked well in your approach?
    b. What challenges have you encountered?
    c. Are there patient factors such as age, comorbid conditions, social support, employment status, etc that would sway you in one direction over another?

2. What have you heard about "shared decision making"?
   [Read after clinician has answered] To make sure we're on the same page, what we mean by shared decision making is a way for patients and clinicians to work together to reach agreement about a health decision involving multiple medically appropriate treatment options. Often there is no clear "best" option for a patient's health condition. Instead the choice depends on patient values and preferences. Now let's talk more about your experience.

3. How often do you use shared decision making in this situation? Does this depend on patient characteristics? Probes:
    a. Age of patient?
    b. Stage of cancer at first presentation/cancer characteristics?
    c. Other health conditions?
    d. Health literacy?
    e. Family history?

4. Have you heard of the term "patient decision aid"? What have you heard about them? Have you ever used a decision aid in the past?
   [Read after clinician has answered] To make sure we are all on the same page, a decision aid is a tool that is designed to help people think about their health choices. It provides information about options, helps people think about what matters to them,

and then helps them make a step by step decision. Decision aids come in different formats such as paper, online, and video.

5. Would a patient decision aid help you communicate with your patients?
    a. How might it help?
    b. How might it hinder the conversation?

6. What information would you like to see in a decision aid about operative vs non-operative management of rectal cancer?
    a. Do you have anything specific that you share with patients, such as standard materials or specific risks that you discuss?

7. What do you think would be the best format for this tool? (eg paper, online, video)
    a. Does this depend on patient characteristics? Probes: Age, health literacy, education?

Thank you so much for your time. We really appreciate this perspective, and we will use what you and others tell us to help improve the way we talk to patients about this decision.
